# The Nauheim Treatment in Heart Cases

**Published:** 1910-06-18

**Authors:** 


### THE NAUHEIM TREATMENT IN HEART CASES.

It is by no means every cardiac patient who will be benefited by the Nauheim treatment. The question as to who should and who should not receive that particular form of treatment is fully discussed by Professor Romberg, of Tübingen, and his conclusions are concisely summarised in the work translated by Dr. Garston which we recently reviewed.

If the stage of severe disturbance of the circulation has already begun, if the heart is already during rest no longer in a position to maintain an approximately normal circulation, if there be continuous and severe dyspnœa during rest, if persistent œdema and effusion into the body cavities be present, then baths and Nauheim gymnastics are no longer suitable for the patient. Equally unsuitable are patients suffering from pronounced angina pectoris and attacks of cardiac asthma, as experience shows that dangerous attacks may be brought on by any increased demand on the heart. Similarly unsuitable also are cases in which fresh disturbances have occurred after manifest over-exertion or after an infectious disease. These are benefited by the treatment only in their later stages.

Great attention should be given to the condition of the vascular system. When considerable arterio-sclerosis is present and the blood-vessels are consequently more or less incapable of adapting themselves to the changing demands made upon them, we must remember that the blood requirements of the body have to be regulated by the heart much more than under normal conditions. In arterio-sclerosis baths and gymnastics make heavy demands on the heart, especially gymnastics, so that in pronounced arterio-sclerosis it is better to avoid resistance exercises entirely. Gymnastics are absolutely contra-indicated in all cases in which chronic nephritis is present. Such patients are liable to violent fluctuations of their vaso-motor tonus. In them the demands on the heart can never be estimated beforehand with any certainty. If, on the other hand, as often happens, cardiac insufficiency is the primary disease and the kidney affection is secondary to it, baths and graduated exercises may be of advantage.

Further, all patients to whom an acceleration of the blood-stream is dangerous are naturally precluded from baths and gymnastics; also persons who have had even a slight attack of apoplexy, persons with distinct cerebral atheroma, and persons who have recently recovered from embolism. If the condition of the brain improves and the state of the heart renders bath treatment desirable, Romberg advises baths of medium, or almost medium, temperature which do not contain carbonic-acid gas, such as plain and thermal brine-baths. Finally, it is scarcely necessary to emphasise the fact that the general condition of the patient should be taken into consideration, and that those who, above everything, require rest, especially neurasthenics, the over-worked, the exhausted, and the very emaciated, should not be put through a severe course of baths or gymnastics, at least at the beginning of the cure.

If weakness of the heart occurs in a person who has led a physically very active life, it is useless to expect benefit from Nauheim gymnastic exercises when the much heavier work required of the heart in his daily occupation has not averted the occurrence of insufficiency. But if none of the contra-indications already enumerated are present, and especially if there is no contributory overstrain, we may well expect good results in cases where the heart of a muscularly weak person fails. Thus heart-weakness in a muscularly weak but corpulent young person with no arterio-sclerosis is very suitable for gymnastic treatment.

Graduated exercises may be more freely prescribed in the after-treatment of cases in which the heart-weakness has ceased to be active than in the treatment of fresh attacks. In the former, careful gymnastic treatment is often a very good introduction to more active bodily exercise, of course only in persons without pronounced arterio-sclerosis, also more especially in young people.

The question may be asked whether there is any real necessity for the physical methods of treatment, or whether the same results cannot be obtained more simply, and with at least equal certainty, by means of drugs. This cannot be answered in a word. The mode of action of each is very different. Drugs enable the heart to utilise more of its available strength. Whether by continued use they increase the sum total of the heart's strength is not known. But baths and gymnastics, by the way they influence the heart increase its strength, just as dumb-bell exercises increase the strength of the muscles of the arm. There are cases in which drugs fail, as, for example, in many forms of cardiac insufficiency occurring in obstinate dilatations of the right side of the heart from mitral stenosis, and when there are lingering remains of overstrain of the heart or of heart-weakness after infective diseases, especially after articular rheumatism. In the first of these, gymnastic exercises, and baths in the last two, render excellent service by increasing the strength of the heart, which is too weak to meet the demands upon it. Augmentation of the working capacity of these hearts can often only be effected by increasing their total strength. As they work they are putting forth all their strength, but that is not sufficient to meet the demands upon them till it is proportionately increased. Drugs by themselves are powerless to extract any more work out of these hearts.

A further question specially raised by August Hoffmann is whether the results of the physical methods of treatment are lasting. Objective statistics on the point are certainly very desirable, but beset with many difficulties. It is always somewhat uncertain to judge from subjective impressions. As far as that is possible it may be said that very satisfactory results can be obtained in this respect when they are used in the restricted manner just indicated. It need hardly be mentioned that all measures taken to strengthen the heart, such as drugs, baths, and graduated exercises, can only be employed with prospect of success if its ordinary work be lightened. This applies especially to physical methods which make increased demands on the strength of the heart and work directly in that way. These demands may so easily augment the ordinary work of the heart that the result may not be the hoped-for improvement, but in certain circumstances direct injury to its strength by overstrain. When drugs are given it is the general rule, and very rightly so, to curtail the patient's work or to order complete rest. By so doing the drugs make no increased demands on the work of the heart, they only make more of its strength active and its work improves with this increase of strength. But it happens daily that patients with heart disease are ordered carbonic-acid baths, gymnastics, or even the Terrain cure without reducing their ordinary duties. That is very undesirable.

To carry out these methods properly the patient requires more rest than he had formerly. The actual amount of rest that should be taken depends on the condition of the patient. Precise rules regarding it cannot be laid down, but it is always better to order too much than to run the risk of overstraining the heart. The necessity of resting the heart holds good in ordering all therapeutic measures. For this reason baths and exercises should not be prescribed together till it is certain that the heart will react to the additional demand on it in the manner desired. After each bath and each gymnastic practice the patient must take ample rest. It is necessary to be specially stringent in ordering rest after overstrain and to persons suffering from the results of overstrain. More or less extensive reduction of the ordinary occupation must likewise be made in cases of obesity with heart insufficiency. Reduction of bodily weight is better effected by dietetic measures. In obesity, moreover, it not infrequently happens that with greater rest and, in certain circumstances, with richer diet than formerly, the patient, to his surprise, loses weight, sometimes very rapidly, as the stronger action of the heart causes excretion of the surplus water previously retained in the body, which in obesity may reach a very significant amount without causing œdema.

